# Transgenic Common Carp Do Not Have the Ability to Expand Populations

**DOI:** 10.1371/journal.pone.0065506

**Published:** 2013-06-07

**Authors:** Hao Lian, Wei Hu, Rong Huang, Fukuan Du, Lanjie Liao, Zuoyan Zhu, Yaping Wang

**Affiliations:** 1 State Key Laboratory of Freshwater Ecology and Biotechnology, Institute of Hydrobiology, The Chinese Academy of Sciences, Wuhan, China; 2 University of Chinese Academy of Sciences, Beijing, China; University of Georgia, United States of America

## Abstract

The ecological safety of transgenic organisms is an important issue of international public and political concern. The assessment of ecological risks is also crucial for realizing the beneficial industrial application of transgenic organisms. In this study, reproduction of common carp (*Cyprinus carpio*, CC) in isolated natural aquatic environments was analyzed. Using the method of paternity testing, a comparative analysis was conducted on the structure of an offspring population of “all-fish” growth hormone gene-transgenic common carp (af*gh*-CC) and of wild CC to evaluate their fertility and juvenile viability. Experimental results showed that in a natural aquatic environment, the ratio of comparative advantage in mating ability of af*gh*-CC over wild CC was 1∶1, showing nearly identical mating competitiveness. Juvenile viability of af*gh*-CC was low, and the average daily survival rate was less than 98.00%. After a possible accidental escape or release of transgenic CC into natural aquatic environments they are unable to monopolize resources from eggs of natural CC populations, leading to the extinction of transgenic CC. Transgenic CC are also unlikely to form dominant populations in natural aquatic environments due to their low juvenile viability. Thus, it is expected that the proportion of af*gh*-CC in the natural environment would remain low or gradually decline, and ultimately disappear.

## Introduction

Transgenic fish technology was first introduced in the 1980s and has been used for the genetic improvement of farmed fish [1], [2]. In the past 20 years, important progress has been made in the research of transgenic fish breeding for the purposes of increasing growth rates. Several fast-growing transgenic fish strains have been bred, showing attractive commercial prospects [3]–[13]. However, there are currently no transgenic fish strains used in commercial production for human consumption.

Food security and the ecological safety of transgenic organisms are of great public concern and represent the last obstacles preventing transgenic fish from entering the market. Compared with food safety assessment, ecological security assessment poses a more difficult task. It is generally believed that fertility and viability are key fitness parameters for evaluating the ecological safety of transgenic fish [14], [15]. In recent years, a number of transgenic fish viability-related traits have been reported, including characteristics of the appetite and feeding behavior of transgenic salmon and common carp [16]–[19], swimming ability [20]–[22], respiratory metabolism characteristics [23], viability under dissolved oxygen and ammonia nitrogen stress [24], [25] and juvenile prey mortality [15], [26], [27]. Studies on transgenic fish fertility mainly involve the transgenic fish gonad index [5], [7], the first sexual maturation time [28], sperm ejaculation volume, sperm motility and mating behavior [29]. Results of comparative studies of single factors of viability and fertility showed that transgenic fish exhibited lower viability and fertility [15], [20], [22], [27]–[29].

However, Muir et al. [30] obtained opposing results in a study on transgenic medaka (*Oryzias latipes*). By assessing a population genetics model based on the findings of fertility and viability of transgenic medaka, they proposed the “Trojan gene hypothesis”. This hypothesis predicts that because of the significant fertility advantage of their transgenic medaka and the low viability of its juveniles, once transgenic medaka were released into a natural aquatic environment, transgenic and wild medaka populations would be extinct within 50 generations [30], [31]. This hypothesis has resulted in a strong public response, but its universality has been questioned by the academic community [32].

We examined and analyzed the fertility and juvenile viability of af*gh*-CC and CC in an isolated pond. The study aimed to obtain fitness parameters of fertility and viability of the two populations in a natural state, thus further assessing the ecological risk for the environment into which af*gh*-CC could be released.

## Materials and Methods

### Experimental Fish

In this study, male parents were used from the same family population of two-year old transgenic fish. Transgenic males were heterozygotes and wild-type males were their full-sib controls, while female parents were three-year wild-type females, provided by the Zhan Dian Breeding Farm of Fisheries Institute of Sciences, Henan Province, China. Ethical approval for the work was obtained from Expert Committee of Biomedical Ethics, Institute of Hydrobiology of the Chinese Academy of Sciences. The Reference number obtained was 091110-1-303.

### Artificial Reproduction and Reproduction Experiment in a Natural Ecosystem

Artificial insemination was conducted using the dry fertilization method. Fertilized eggs were hatched in culture dishes at an average water temperature of 18.5°C. The fertilization rate was calculated when fertilized eggs developed into the segmentation phase and myocomma appeared. The hatching rate was calculated when juveniles emerged from the membrane (0 days).

The reproduction experiment was conducted in an outdoor isolated -pond as a natural ecosystem. The pond had an area of approximately 1400 m^2^ and a depth of 1.5–2.0 m. Six transgenic males (T1–T6), six wild-type males (N1–N6) and six wild-type females (W1–W6) constituted a natural reproductive population. The reproductive population was put into the experimental pond one month before the breeding season to perform natural spawning. Parents were removed after mating which was continued for eight days. Fertilized eggs and juveniles hatched and grew under the natural conditions of the pond. During the experiment, no human intervention, such as enriching oxygen, changing water or feeding were conducted to fully simulate the natural process of breeding. Forty-day-old juvenile offspring were used for paternity testing and PCR detection of transgenes.

### DNA Sample Preparation and Transgene Detection

Genomic DNA of fin rays or fry were prepared using the phenol-chloroform extraction method. The PCR reaction (10 µL) contained 1 µL genomic DNA (50 ng/µl), 1 µL 10×buffer (containing Mg^2+^), 0.5 µL Taq DNA polymerase (1 U/µL), 0.4 µL dNTPs (2.5 mmol/L each), 0.4 µL each of forward and reverse primers (10 µmol/L) and 6.3 µL ddH_2_O. The thermocycling program consisted of pre-denaturation at 94°C for 3 min, 30 cycles of amplification (denaturation at 94°C for 30 s, annealing at 58°C for 30 s and extension at 72°C for 40 s), and a final extension at 72°C for 5 min. PCR products were detected on a 1% agarose gel by electrophoresis.

### Microsatellite Marker Screening and Genotyping

Thirty candidate marker loci were selected from carp microsatellite MFW series [40], KOI series [41] and HLJ series [42]. The 18 parent fish described above were taken as the detection group for microsatellite marker screening. The PCR reaction system (25 µL) contained 1 µL genomic DNA (50 ng/µL), 2.5 µL 10×buffer (including Mg^2+^), 0.8 µL dNTPs (2.5 mmol/L each), 0.8 µL each of forward and reverse primers (10 µmol/L), 1 µL Taq DNA polymerase (1 U/µL) and 18.1 µL ddH_2_O. The PCR reaction program consisted of pre-denaturation at 94°C for 3 min, 30 cycles of amplification (denaturation at 94°C for 30 s, annealing at 58°C for 30 s and extension at 72°C for 40 s), and a final extension at 72°C for 5 min. Genotyping of PCR products was detected using the LI-COR 4300 DNA gel electrophoresis system. The polymorphic information content (PIC) and heterozygosity (H) at each locus were analyzed using POPGEN (Version 1.32) software. Nine appropriate SSR loci were screened for paternity testing (Table 1). Marker genotyping of paternity testing samples was conducted using the same method.

**Table 1:** Primers used for PCR amplification.

Locus	Primer^+^	Primer−	H	PIC
MFW1	mGTCCAGACTGTCATCAGGAG	GAGGTGTACACTGAGTCACGC	0.880	0.867
MFW9	mGATCTGCAAGCATATCTGTCG	ATCTGAACCTGCAGCTCCTC	0.788	0.763
MFW11	mGCATTTGCCTTGATGGTTGTG	TCGTCTGGTTTAGAGTGCTGC	0.799	0.777
MFW15	mCTCCTGTTTTGTTTTGTGAAA	GTTCACAAGGTCATTTCCAGC	0.853	0.836
HLJ38	mCACAGAACGCATCAGTAA	TGTAAACCTTCAACCTCC	0.860	0.844
MFW19	mGAATCCTCCATCATGCAAAC	GCACAAACTCCACATTGTGCC	0.838	0.817
MFW18	mGTCCCTGGTAGTGAGTGAGT	GCGTTGACTTGTTTTATACTAG	0.708	0.665
MFW26	mCCCTGAGATAGAAACCACTG	CACCATGCTTGGATGCAAAAG	0.855	0.837
MFW29	mGTTGACCAAGAAACCAACATGC	GAAGCTTTGCTCTAATCCACG	0.726	0.728
PII- Pc	CATTTACAGTTCAGCCATGGCTAGA	AGCACCACCGACAACAGCACTAATG	–	–

NOTE:+“m” represents the M13 sequence (CACGACGTTGTAAAACGAC).

### Paternity Testing and Statistical Analysis

Paternity testing was conducted using Cervus 2.0 software. Allele frequencies (P), exclusion probabilities (PE), cumulative chance of exclusion (CCE), natural logarithm of likelihood ratio (LOD) values and delta values at each locus were calculated. The likelihood ratio of each parental candidate [43] was counted. Delta values of assumed parents were calculated by a simulation program to ensure assessment of parentage with high statistical confidence [44]. Significant differences between populations were detected by t-test or chi-square test.

## Results

### Genetics and Reproductive Biology Characteristics of Transgenic CC

**Figure 1:**
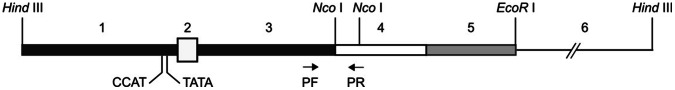
pCAgcGH structure diagram. 1: carp *β-actin* gene 5′-flanking sequence; 2: carp *β-actin* gene first exon; 3: carp *β-actin* gene first intron; 4: grass carp *GH* gene sequence; 5: grass carp *GH* gene 3′-flanking sequence; 6: plasmid pUC118. PF and PR indicate PCR primers of transgenes.

Transgenic CC carried recombinant af*gh*, which is the grass carp growth hormone gene driven by the CC β-actin promoter (pCAgcGH) (Figure 1). P0 transgenic males obtained by microinjection were hybridized with wild-type females to obtain F1 transgenic fish heterozygous groups [10]. Fast-growing F1 transgenic heterozygous males were selected to hybridize with wild-type females to screen for a fast-growing transgenic fish F2 family whose unit points were integrated. In this family group, transgenes showed Mendelian segregation (1∶1) (transgenic fish:non-transgenic fish), where transgenic fish were transgenic heterozygotes. We conducted selfing of the F2 transgenic fish for the screening of the homozygous transgenic family; F2 transgenic males hybridized with wild-type females to establish F3 transgenic fish-segregated populations. Similarly, transgenic fish were transgenic heterozygotes. Transgenic fish and non-transgenic fish showed 1∶1 segregation. Transgenes were passed between generations in a Mendelian way.

Experimental results of artificial fertility-hatching showed that reproductive biology characteristics of F3 transgenic fish were similar to those of the controls. Three F3 transgenic males and three full-sib non-transgenic males were used to fertilise eggs of the same wild-type female, respectively. Average fertilization rates of transgenic fish and non-transgenic fish were 90.37±3.48% and 92.82±5.24%, respectively (*t = *0.68; *P* = 0.54; *df* = 4). Their average hatching rates were 85.60±8.05% and 85.20±10.36%, respectively (*t* = −0.05; *P* = 0.96; *df* = 4). Under laboratory conditions, no significant differences were found in the fertility between transgenic and non-transgenic fish (Table 2). Transgene detection by PCR was conducted on the offspring population of transgenic males. In offspring populations of three transgenic males, ratios of transgenic fish were 48% (48/100) (χ^2^ = 0.08; *P* = 0.78), 47% (47/100) (χ^2^ = 0.18; *P* = 0.67) and 51% (51/100) (χ^2^ = 0.02; *P = *0.89). The segregation of transgenes in the offspring population was consistent with a Mendelian segregation ratio of 1∶1 (Table 2). These results indicate that transgenes passed steadily between generations of transgenic fish, that sperm carrying transgenes and their controls had the same level of fertilization ability, and they did not impact on the early development of embryos.

**Table 2:** Fertilization, hatching rates and transgene segregation.

	Non-transgenic males			Transgenic males			*P*
Fertility (%)	87.05	94.15	97.27	94.15	89.66	87.31	>0.05
Hatchability (%)	73.38	92.68	89.55	94.15	84.48	78.17	>0.05
Segregation (%)	–	–	–	48	47	51	>0.05

### Composition of the Natural Fertility Population and their Offspring Population

From the segregated populations of two-year old F3 transgenic fish described above, six transgenic males (T1–T6) and six full-sib non-transgenic males (N1–N6), plus six wild-type females aged three years (W1–W6) were selected to constitute a fertility population including six females and 12 males. The weight distribution of transgenic males was 1.31–2.63 kg, and average weights of three small individuals (T1–T3) and three large individuals (T4–T6) were 1.49±0.16 kg and 2.55±0.09 kg, respectively. Non-transgenic males showed a smaller weight distribution (0.80–1.17 kg), and the average weight was 0.98±0.15 kg; the weight distribution of wild-type females was 3.50–4.84 kg, and the average weight was 4.22±0.53 kg (Table 3).

**Table 3:** Number of offspring of all parental combinations.

Wild-type females (body weight, kg)	Non-transgenic males						Transgenic males					
	N1 (0.87)	N2 (0.87)	N3 (0.80)	N4 (1.05)	N5 (1.17)	N6 (1.10)	T1 (1.62)	T2 (1.31)	T3 (1.54)	T4 (2.46)	T5 (2.56)	T6 (2.63)
W1 (4.08)	9	15	16	20	44	19	12	12	9	5	16	8
W2 (3.89)	21	10	14	12	18	21	11	8	22	18	22	5
W3 (3.50)	9	20	8	22	33	17	19	6	13	10	9	9
W4 (4.14)	15	7	18	32	37	33	10	15	5	13	17	11
W5 (4.84)	35	27	18	10	34	13	8	19	23	31	8	13
W6 (4.84)	7	10	14	19	22	15	8	8	15	9	12	5

Six females and 12 males described above were released into an isolated pond before the reproductive season. In late March, experimental fish started spawning, and spawning was continued for eight days. After spawning, reproductive parents were removed from the pond, fertilized eggs and fry were naturally hatched and grown in the pond without human intervention. After 40 days, 1200 juveniles with an average weight of 0.28±0.03 g were randomly sampled from the offspring population for paternity testing.

Marking and typing were conducted on nine SSR loci. Complete SSR typing data were obtained from 1138 juvenile samples. The paternity testing results showed that these 1138 offspring were from 72 parental combinations (12×6), that is, six females successfully mated with 12 males and produced offspring populations ranging from 5 to 44 offspring (Table 3).

### Juvenile Viability of af*gh*-CC Descendants

According to the results of paternity testing, six transgenic males in the testing samples produced 444 offspring (Table 3). Transgene PCR results showed 54 transgenic fish and 390 non-transgenic fish. In offspring populations of transgenic males, the transgenic fish ratio was 12.16%, strongly deviating from the theoretical value of 50% (Table 4). The juvenile viability of descendants of af*gh*-CC was significantly lower than that of their non-transgenic full-sib controls (χ^2^ = 148.37; *P* = 0.00). During the 40–48 day experiment, the relative viability of transgenic juveniles was 13.85%, and the average daily relative viability was 0.98.

**Table 4:** Number of offspring form male parents.

	N1	N2	N3	N4	N5	N6	T1	T2	T3	T4	T5	T6
Transgenic offspring	–	–	–	–	–	–	9	8	12	9	13	3
Non-transgenic offspring	96	89	88	115	188	118	59	60	75	77	71	48
Sum	96	89	88	115	188	118	68	68	87	86	84	51

In the offspring population of transgenic males at different sizes, ratios of transgenic fish showed no significant difference. Among 223 offspring of small transgenic males (T1–T3), there were 29 transgenic fish, and the ratio of transgenic fish was 13.00%. Among 221 offspring of large transgenic males (T4–T6), there were 25 transgenic fish, and the ratio of transgenic fish was 11.31% (Table 4). The size of transgenic males did not impact on their offspring viability (χ^2^ = 0.30; *P* = 0.59).

### Effect of Body Size on Reproductive Success in afgh-CC Males

**Figure 2:**
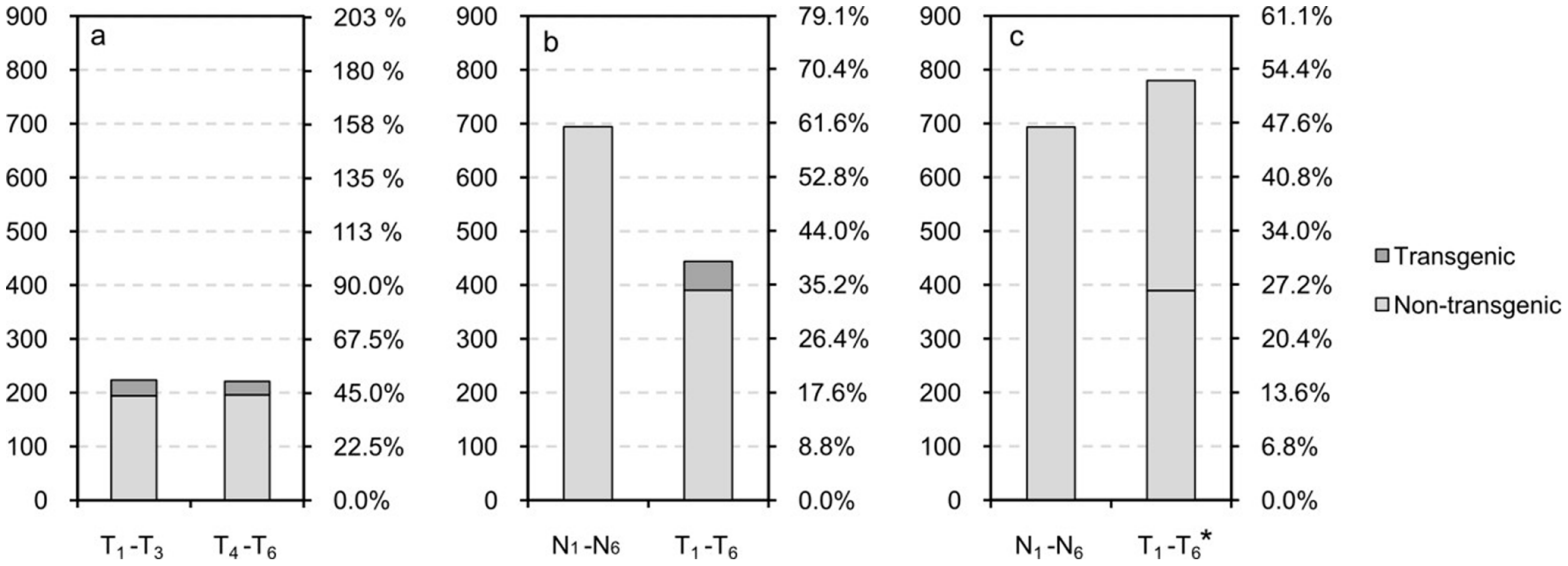
Number and ratio of each offspring population and the significance test between offspring populations. a: T1–T3 are the numbers of offspring of small transgenic males, T4–T6 are the numbers of offspring of large transgenic males; b: N1–N6 are the numbers of offspring of wild-type males, T1–T6 are the numbers of offspring of transgenic males; c: N1–N6 are the numbers of offspring of wild-type males, T1–T6* are the numbers of offspring of transgenic males after correction. Left vertical axis shows the number of individuals; right vertical axis shows the ratio of offspring populations.

Offspring populations of transgenic males (T1–T6) at different sizes were compared. The numbers of offspring from small males (T1–T3) and large males (T4–T6) were 223 and 221, respectively (Table 4), and ratios in offspring populations of transgenic males were 50.23% and 49.77%, respectively. A chi-square test showed that the number of offspring of T1–T3 and T4–T6 were not significantly different (χ^2^ = 0.01; *P = *0.95) (Figure 2a).

Since no significant differences were found in the offspring viability, population size and composition of T1–T3 and T4–T6, the offspring population from T1–T6 was compared with that from non-transgenic males (N1–N6). The number of offspring from T1–T6 was 444, and that from N1–N6 was 694 (Table 4), while their ratios of offspring population were 39.01% and 60.09%, respectively. The number of offspring from T1–T6 was significantly lower than that from N1–N6 (χ^2^ = 27.80; *P = *0.00) (Figure 2b).

Taking into account the high mortality of transgenic juveniles, the number of surviving offspring of transgenic males could not accurately reflect the number of fertilized eggs, and a direct comparison of the number of surviving offspring would underestimate the fertility of transgenic males. Therefore, twice the number of non-transgenic offspring of transgenic males was taken as the corrected value of the number of offspring from transgenic males; that is 780 (2×390) offspring. Results of the comparison after this correction showed that the ratio of offspring from T1–T6 in the population was 52.92%, and that from N1–N6 was 47.08% (Figure 2c). Based on this finding, the comparative advantage in mating ability of transgenic carp to wild-type carp was 1∶1, with no significant difference between the two groups (χ^2^ = 0.18; *P = *0.67).

## Discussion

The “Trojan gene hypothesis” proposed by Muir et al. [30] assumes that transgenic males would be significantly larger than control males, thus holding a dominant position when competing for a mate. In contrast, the hypothesis states the viability of offspring from transgenic fish is significantly lower than that of wild-type fish. The combination of these two factors would lead to the extinction of the entire population, and transgenes show the so-called “Trojan effects” [30], [31]. In this study of af*gh*-CC and wild CC, paternity testing of 1138 offspring showed that transgenic and wild-type males, regardless of their individual sizes, were successfully involved in breeding, including small males. This result differs substantially from the situation observed in medaka [31]. Carp follow the reproductive strategy of “group spawning” and have the characteristic of batch spawning (batch spawning and promiscuity of carp account for this similarity) [33]–[35]. The different reproductive strategies of carp and medaka are probably the main reason for the different results described above. These results suggest that the role of male size in competitive mating arenas and its effects on the structure of their offspring populations is different in fish employing different reproductive strategies. Large individuals do not result in an advantage in fertility. In a study of af*gh*-modified Atlantic salmon (*Salmo salar*), even the opposite phenomenon was found, with the fertility of large transgenic fish significantly decreased [36]. Based on findings from different transgenic fish species, it is hard to draw a general conclusion on the ecological risk assessment of transgenic fish. Targeted case analyses are still required for the ecological risk assessment of transgenic fish.

Male fertility is an embodiment of the competitive ability of mating and sperm. It is essentially the ability of males to fertilize eggs. It is difficult to accurately observe competitive breeding behavior under natural conditions. In this experiment, the number of surviving offspring of males was used to calculate the number of fertilized eggs, further calculating the relative fertility advantages of transgenic and wild-type males. As described in the results section of this article, because of the significant difference in the viability between transgenic and wild-type juveniles, the number of surviving transgenic juveniles could not truly reflect the number of fertilized eggs of transgenic males. A direct comparison of the number of surviving offspring between transgenic and wild-type males would underestimate the actual fertility of transgenic males. Artificial insemination-incubation experiments have shown that heterozygous transgenic males produce an equal number of transgenic and wild-type sperms with the same fertilization capacity. Therefore, it would be more accurate to estimate the total number of fertilized eggs by using the number of wild-type surviving offspring of transgenic males. The fertility comparison indicated that the fertility ratio of transgenic males to wild-type males was 1∶1, showing no significant differences. A larger body size of transgenic carp did not confer any advantage in breeding competitiveness.

Fast-growing transgenic fish generally exhibit lower juvenile viability [8], [5], [37], [38]. The increased metabolism of fast-growing transgenic fish may be the main cause of high juvenile mortality [9]. Juvenile viability differs between different transgenic fish species. The daily viability of 3-day-old transgenic medaka reached 91.5–93.0% [37], while all juveniles of transgenic coho salmon died within a few weeks due to food shortage [38]. In the present study, the viability of 40–48-day-old transgenic carp was 13.8%, and the average daily viability was 98.0%. Because most transgenic juveniles die at an early stage of initial feeding, the daily viability in the early stage will be lower. According to the ecological risk assessment model of transgenic fish established by Muir *et al.* [30], [39], if the relative fertility advantage was 1∶1 and the daily survival rate was 98.00% or less, transgenic fish populations would survive. Transgenic carp differ from transgenic medaka in that they do not present “Trojan effects” leading to the extinction of the population.

### Conclusions

Our results show that in a natural aquatic environment, fast-growing transgenic common carp have no advantages in fertility, and that their juvenile viability is low. We suggest that transgenic carp escaped or released into the natural aquatic environment would be unable to monopolize the eggs of natural CC populations, thus leading to population extinction. Moreover, transgenic CC are incapable of forming dominant populations in natural aquatic environments due to their low juvenile viability. Therefore, we predict that the ratio of transgenic CC in natural populations would remain at low levels or gradually decline, and ultimately disappear.
